# Recruitment maneuvers in patients with acute respiratory distress syndrome: a systematic review and metanalysis

**DOI:** 10.31744/einstein_journal/2024RW0372

**Published:** 2024-11-26

**Authors:** Alice Cardoso de Jesus, Arthur Marchesini de Figueiredo, André Luiz Lisboa Cordeiro

**Affiliations:** 1 Centro Universitário Nobre Feira de Santana BA Brazil Centro Universitário Nobre, Feira de Santana, BA, Brazil.

**Keywords:** Acute respiratory distress syndrome, Mortality, Oxygenation

## Abstract

**Objective:**

To systematically review the effects of recruitment maneuvers on patients with acute respiratory distress syndrome.

**Methods:**

This systematic review and meta-analysis using the PICO methodology with keywords (respiratory distress syndrome, recruitment maneuvers, lung recruitment, acute respiratory distress syndrome, alveolar recruitment, and adult acute respiratory distress syndrome). Studies involving patients >18 years, regardless of sex, with acute respiratory distress syndrome, mechanically ventilated for at least 24 h, published in English, Portuguese, and Spanish, with no year restrictions, were included. Studies that combined recruitment maneuvers with other techniques and those conducted in animals were excluded. Boolean operators "AND" and "OR" were used.

**Results:**

Fifteen studies were included. The recruitment maneuver proved to be effective in oxygenating patients (mean difference=45.05 mmHg (95% confidence interval (95%CI): 31.37-58.74)), but there was no statistically significant difference in the rate of mortality OR=0.89 (95%CI=0.74-1.08) and barotrauma RR=0.93 (95%CI=0.56-1.54).

**Conclusion:**

Recruitment maneuvers should not be used routinely in the care of patients with acute respiratory distress syndrome, but it is a good rescue strategy when other methods fail to improve oxygenation.

**Prospero database registration:**

(www.crd.york.ac.uk/prospero) under ID CRD42021227231.

## INTRODUCTION

Acute respiratory distress syndrome (ARDS) is an inflammatory disease characterized by the most severe form of lung injury.^(1)^ Ventilatory support has been used to improve patient survival.^(2)^ Recruitment maneuvers (RMs) aim to recruit collapsed alveoli, increasing the lung area available for gas exchange.^(3)^

Acute respiratory distress syndrome has a high incidence, affecting about 10% of patients admitted to intensive care units (ICUs) worldwide, with over 20% requiring ventilatory support. Mortality rates are high, ranging between 30 and 40%.^(4)^

Due to the high mortality rate, prolonged length of stay, decreased functional capacity and quality of life^(1,2)^ it is essential to combine mechanical ventilation with new treatment approaches for ARDS, such as positive end-expiratory pressure (PEEP) titration, prone position,^(5)^ and the alveolar (RM), which is an open lung strategy to improve ventilation.^(6)^

Evidence shows that performing RM in patients with ARDS improves prognosis,^(1,2)^ length of hospital stay, oxygenation,^(7)^ and decreases barotrauma risks,^(8)^ as it allows the alveoli to open^(6)^ by increasing transpulmonary pressure, consequently improving blood oxygenation.^(8)^

## OBJECTIVE

The main objective of this paper was to review the evidence of alveolar recruitment maneuvers on mortality, and also on barotrauma and oxygenation.

## METHODS

### Protocol and register

This systematic review was conducted in accordance with the Preferred Reporting Items for Systematic Reviews and Meta-Analyses guidelines.^(9)^

### Eligibility criteria

The PICOS^(10)^ strategy was used, where the study population included patients with ARDS, which was the RM, compared to patients who received standard treatment. The primary outcome was mortality, and the secondary outcomes were barotrauma and oxygenation. Randomized clinical trials were conducted in English, Portuguese, and Spanish, without restricting the year.

### Research sources

We conducted computer-based research by consulting the Web of Science, SCOPUS, EMBASE, PubMed, Physical Therapy Evidence Database (PEDro), and the Cochrane Central Register of Systematic Reviews. We also searched reference lists from previous systematic reviews and clinical trials that were eligible. The search for articles was ended in April 2021.

### Search

The search was based on the previously described PICOS strategy and the Boolean operators "AND" and "OR". Descriptors were used for the population respiratory distress syndrome, adult acute respiratory distress syndrome, human respiratory distress syndrome, acute chest syndrome, respiratory distress syndrome, adult respiratory distress syndrome, shock lung, ARDS, adult ARDS, and human ARDS. RM, alveolar RM, alveolar recruitment, pulmonary RM, pulmonary volume recruitment, and pulmonary recruitment were used. Outcomes included mortality, in-hospital mortality, ICU mortality, barotrauma, and oxygenation. For the study design, descriptors used were randomized controlled trials, clinical trials, and controlled trials. The search strategy is shown in Supplementary Material.

### Study selection

Randomized clinical trials involving patients with ARDS who were intubated and mechanically ventilated in the ICU for at least 24 h were included in this systematic review. Studies involving adults of both sexes were included. ARDS was classified, according to the Berlin definition, as mild (PaO_2_/FIO_2_ between 300-201), moderate (PaO_2_/FIO_2_ between 200-101) and severe (PaO_2_/FIO_2_ ≤100), in patients using PEEP ≥5 or 10cmH_2_O, with pulmonary edema of non-cardiogenic origin and bilateral opacities on radiography.^(11)^ RMs were considered as any transient increase in airway pressure aimed at restoring or improving lung aeration.

Studies that combined RM with other techniques, those that performed RM in both groups, or those performed in animals were excluded.

### Data collection process

To extract the selected articles, titles (first step), abstracts (second step), and complete readings (third step) were verified. Exploratory reading of the selected studies was then conducted, followed by a selective and analytical reading. The data extracted were summarized by authors, journals, years, titles, and conclusions to obtain important information.

Two independent reviewers assessed the methodological quality of the included studies. When there was a divergence, the article was read in its entirety for re-evaluation. If a disagreement persisted, a third reviewer assessed and made a final decision.

### Data items

Two authors independently extracted data using standard data extraction, considering (1) study population details, such as mean age, sex, sample, and diagnosis; (2) intervention performed (PEEP values used, intervention time, and use of protocols); (3) follow-up details; (4) loss to follow-up; (5) outcome measures; and (6) results presented.

### The quality of each study

The bias risk of the selected papers was classified as low, uncertain, or high based on the criteria established by the Cochrane Collaboration tool.^(12)^

### Synthesis of results

Heterogeneity was evaluated using the χ^2^ test and I^2^ statistic. This statistic illustrates the percentage of variability in effect estimates due to heterogeneity rather than sampling error.

### Statistical assessment

The mean differences (MD) between the groups and their respective 95% confidence intervals (95%CI) were calculated and used to quantify the effect of continuous outcomes. For meta-analyses in which the studies used the same scales, the results were presented as MD and 95%CI. The effects were calculated using the standardized mean difference (SMD) and 95%CI. The effect size of the interventions was defined as small (MD<10% of the scale or SMD<0.4), moderate (MD=10%-20% of the scale, SMD=0.41-0.7), or large (MD >20% of the scale, or SMD >0.7).

## RESULTS

### Selection and characteristics of studies

**Figure 1:**
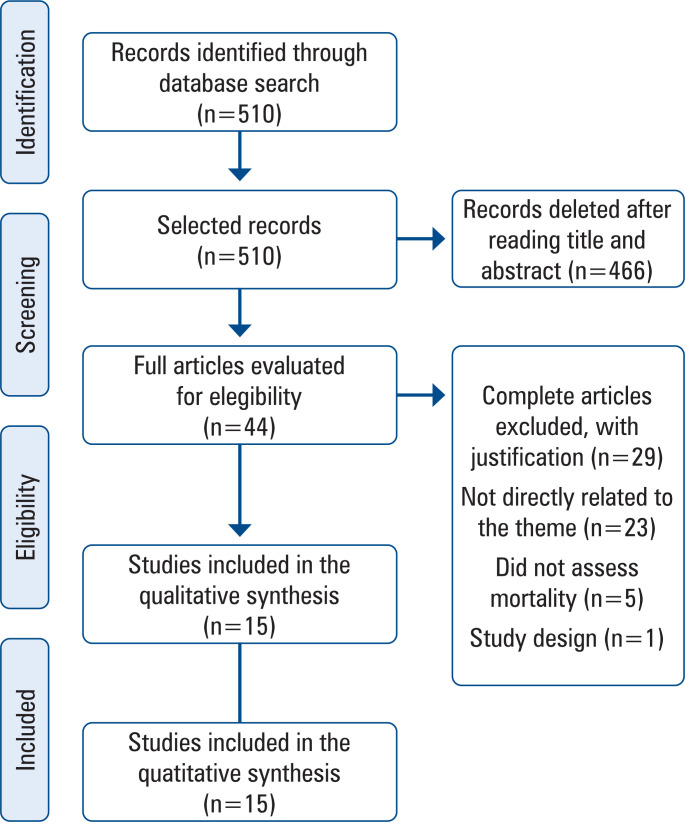
Select process

The database search yielded 510 articles (Figure 1), with 466 initially excluded based on title screening, then from the 44 evaluated from reading the abstracts, 23 were considered not directly related to the study theme. Twenty-one articles were selected for full reading, of which five were excluded for not evaluating mortality and one for inadequate design. This systematic review included 15 articles that met the inclusion criteria.

### Methodological Quality Results

According to the Cochrane Collaboration's Randomized Clinical Trials Risk of Bias Assessment Tool^(12)^ ten studies were classified as having an uncertain or low risk of bias. Most of the selected studies were classified as having a low risk of bias (when most of the information provided had a low risk of bias). The criteria evaluated using the risk of bias assessment tool for randomized clinical trials and the classification obtained by each study are presented in table 1.

**Table 1:** Classification of articles according to the risk of bias

Studies	Random sequence generation	Allocation concealment	Blinding of participants and professionals	Blinding of outcome evaluators	Incomplete outcomes	Selective outcome reporting	Other sources of bias
Amato et al.^(13)^ (1998)							
ART^(14)^ (2017)							
Chung et al.^(15)^ (2017)							
Constantin et al.^(16)^ (2019)							
Hodgson et al.^(17)^ (2011)							
Hodgson et al.^(18)^ (2019)							
Huh et al.^(19)^ (2009)							
Kacmarek et al.^(20)^ (2016)							
Kung et al.^(21)^ (2019)							
Lam et al.^(22)^ (2019)							
Liu et al.^(23)^ (2011)							
Maede et al.^(24)^ (2008)							
Wang et al.^(25)^ (2007)							
Xi et al.^(26)^ (2010)							
Yu et al.^(27)^ (2017)							

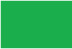
 Low risk of bias

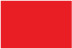
 High risk of bias

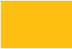
 Uncertain bias risk

### Participants

A total of 1,608 patients received interventions in the included studies. Their ages ranged from 33^(13)^ to 67^(15)^ years, with 1.019 (63.3%) male participants. Additional data are shown in table 2.

**Table 2:** Summary of characteristics of the analyzed studies

Study (Author/year)	Country	Sample	Participants	Interventions		Measurements	Results
				Intervention	Controle		
Amato et al.^(13)^ (1998)	Brazil	53	Patients in the disease process associated with ARDS, with a lung injury score of 2.5 or more and a pulmonary artery pressure of less than 16mmHg	The recruitment maneuver was performed in CPAP mode from 35 to 40cmH2O for 40s	PaCO_2_ 35 to 38 mmHg, FiO_2_ <60%, VC 12ml/kg, flow 50 to 80l/min, inspiratory pause of 0.4s and RR of 10 to 24ipm	Mortality within 28 days, in-hospital mortality, barotrauma	After 28 days, 11 of 29 patients in the Intervention Group died, compared with 17 of 24 in the Control Group (p<0.001). The rate of barotrauma was lower in the intervention group (7% *versus* 42%, p=0.02).
ART^(14)^ (2017)	Brazil	1010	Patients over 18 years old admitted to an intensive care unit, mechanically ventilated for less than 72 hours with ARDS	The recruitment maneuver was performed in PCV mode, with PEEP of up to 35cmH20	Conventional approach	Mortality within 28 days, ICU and in-hospital mortality, mortality within 6 months and barotrauma within 7 days	Mortality rates were higher in the Experimental Group than in the Control Group. The values of the PaO_2_/FiO_2_ ratio were also higher in the Intervention Group.
Chung et al.^(15)^ (2017)	Taiwan	24	Patients who meet the criteria for the diagnosis of ARDS	Participants performed a recruitment maneuver in PCV mode, with PEEP of up to 40cmH2O for up to 40s	Patients did not undergo recruitment maneuver	Oxygenation parameters, ICU and in-hospital mortality	PaO_2_/FiO_2_ increased in the Intervention Group and there was no change in the Control Group (p=0.02). There was no significant difference between the groups in in-hospital and ICU mortality.
Constantin et al.^(16)^ (2019)	France	400	Patients diagnosed with ARDS <12h, PaO_2_/FiO_2_ ≤200 and a PEEP ≥5cmH2O	The recruitment maneuver was performed in CPAP mode, with 35cmH2O for at least 30s	Patients ventilated with PEEP titration based on the PEEP/FiO2 table	Mortality within 90 days, mortality within 28, 30, 180 and 365 days, mortality in the ICU, and number of patients with barotrauma associated with ventilation	By day 90, 56 patients in the Control Group had died compared to 53 patients in the Intervention Group. (HR=0.96; 95%CI=0.66-1.4; p=0.84). There was no interaction between death and recruitment maneuver in both groups (HR 0.9; 0.60-1.3; p=0.64). The rate of barotrauma did not generate a significant difference between groups (p=0.63).
Hodgson et al.^(17)^ (2011)	Australia	20	Patients diagnosed with ARDS	The recruitment maneuver was performed in PCV mode, with PEEP of up to 40cmH20 for 2 min	Patients ventilated according to the ARDSNet protocol	PaO_2_/FiO_2_ ratio and in-hospital mortality	PaO_2_/FiO_2_ was higher in the intervention group in the first 24 h and within 7 days. There was no significant difference in in-hospital mortality.
Hodgson et al.^(18)^ (2019)	Australia	113	Patients admitted to the ICU mechanically ventilated for less than 72 hours with a diagnosis of moderate to severe ARDS	Patients were recruited in PCV mode, with PEEP of up to 40cmH2O for 2 min	Ventilated in volume-controlled mode with low PEEP, VT of 6 ml/kg and plateau pressure ≤30cmH2O	PaO_2_/FiO_2_ ratio, mortality, barotrauma rate	No significant differences were found in mortality or barotrauma rate. PaO_2_/FiO_2_ was higher in the Intervention Group
Huh et al.^(19)^ (2009)	South Korea	57	Patients admitted to an intensive care unit diagnosed with ARDS	The recruitment maneuver was performed in VCV mode, with PEEP of up to 25cmH2O for 30s	Patients ventilated with PEEP titration based on the PEEP/FiO2 table	PaO_2_/FiO_2_ ratio, mortality within 28 and 60 days	Oxygenation significantly improved in both groups. The mortality rate was not significantly different
Kacmarec et al.^(20)^ (2016)	USA	200	Adult patients admitted to the ICU and meeting the criteria of the European-American Consensus Conference for ARDS	The recruitment maneuver was performed in PCV mode with PEEP of 35 to 45cmH2O	Patients ventilated according to the ARDS Network protocol	Mortality within 60 days, in-hospital and intra-ICU mortality, incidence of barotrauma	No statistical differences were found in mortality within 60 days between both groups (n=28, 29% OLA *versus* n=33, 33% ARDSNet) (p=0.18). There was an improvement in oxygenation in the Intervention Group. Barotrauma events were not reported
Kung et al.^(21)^ (2019)	Taiwan	254	Patients admitted to the ICU with ARDS on mechanical ventilation for less than 72 hours	The recruitment maneuver was performed in PCV mode, where PEEP was increased until the peak pressure reached a value of 50cmH2O and maintained for 2 min	They performed protective ventilation	Mortality within 28 and 60 days, barotrauma rate and PaO_2_/FiO_2_ ratio	There was no significant difference in mortality up to 28 and 60 days. The rate of barotrauma was also not significant between groups. Oxygenation was significantly higher on days 1 and 3
Lam et al.^(22)^ (2019)	Vietnam	66	Moderate to severe burn patients presenting ARDS	The recruitment maneuver was performed in PCV mode, with a maximum PEEP of 45cmH20 for 2 minutes	Ventilated according to the ARDS Net protocol	PaO_2_/FiO_2_ ratio and mortality rate	Significantly greater increase in the PaO_2_/FiO_2_ ratio in the Intervention Group during the first 5 days. The mortality rate at 7 days after initiation was lower in the Intervention Group (24.2% *versus* 63.6%, p<0.01), but not after 14 and 28 days
Liu et al.^(23)^ (2011)	Taiwan	100	Patients with ARDS, with PaO_2_/FiO_2_ <250mmHg	The recruitment maneuver was performed with a PEEP of 35cmH2O, maintaining for 2 min	Protective Ventilation Strategy	In-hospital mortality within 28 days	Unable to collect data
Maede et al.^(24)^ (2008)	Canada	985	Patients admitted to the intensive care unit with acute lung injury	Maneuver and recruitment was performed in PCV mode, where he maintained an airway pressure of 40cmH2O for 40 s	Ventilated with conventional levels of positive pressure	In-hospital mortality, during mechanical ventilation in the ICU and within 28 days. Barotrauma rate and PaO_2_/FiO_2_ ratio	No significant differences were found in mortality or barotrauma rate. Oxygenation was higher in the Intervention Group over the days
Wang et al.^(24)^ (2007)	China	28	Patients with ARDS, with PaO_2_/FiO_2_ <200mmHg	They performed CPAP with 35cmH2O for 35s	They used the PEEP/Fio2 table of the ARDS Net group	PaO_2_/FiO_2_ ratio and mortality within 28 days	Unable to collect data
Xi et al.^(26)^ (2010)	China	110	Patients with ARDS, with PaO_2_/FiO_2_ <200mmHg	The recruitment maneuver was performed in CPAP mode with 40cmH2O for 40s	Titration of PEEP for a PaO2 >60mmHg and FiO2 <0.6	Mortality in the ICU, mortality within 28 days and incidence of barotrauma	There was a significant increase in PaO_2_/FiO_2_ in both groups. There were no significant differences in in-hospital mortality (41.8% *versus* 56.4%, p=0.13) and ICU mortality in the Intervention Group was significantly lower. No Barotrauma reports
Yu et al.^(27)^ (2017)	China	74	Patients with ARDS, with PaO_2_/FiO_2_ ≤300mmHg	The recruitment maneuver was performed in SIMV mode, with PEEP increment up to a Peak Pressure of 45cmH20, soon after, they reduced PEEP to 15cmH20 and maintained for 10 min	Uninformed	Mortality within 28 days, ICU mortality, in-hospital mortality and barotrauma	There was no significant difference in the mortality rate or in the barotrauma rate between the groups. PaO_2_/FiO_2_ was significantly higher in the Intervention Group

### Intervention

All studies included in this review opted for RMs, and periodically assessed mortality, and patient outcomes. Oxygenation (PaO_2_/FiO_2_) was assessed by arterial blood gas analysis, and barotrauma was assessed by chest X-ray. Regarding the parameters used in the maneuver, there were some differences between the studies (Table 2). Some studies used the continuous positive airway pressure mode, and others used the controlled pressure mode, and the time of application of the technique ranged from 35 to 40 seconds.

### Mortality

**Figure 2:**
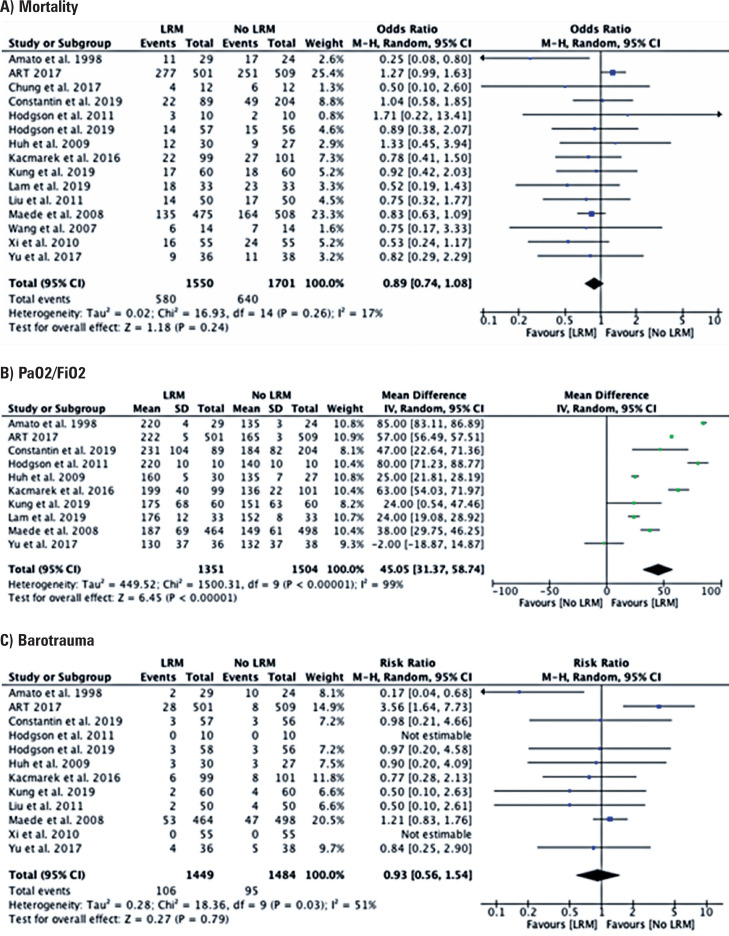
Graphs of meta-analysis results

Fifteen studies^(13–27)^ analyzed the impact of RM on mortality rate. For the meta-analysis of this comparison, a random-effects model was used (I²=17%, df=14, p=0.26) in which there was no statistically significant difference between the groups in the comparison between the RM and the control (odds ratio=0.89; 95%CI=0.74-1.08) (Figure 2A).

A) Comparison of the recruitment maneuver with control over the mortality rate. Values shown are the mean effects (difference between means) and 95% confidence intervals. The mean effect was calculated using a random-effects model B) Comparison of the recruitment maneuver with control over oxygenation. Values shown are the mean effects (difference between means) and 95% confidence intervals. The mean effect was calculated using a random-effects model. C) Comparison of the recruitment maneuver with control over the risk of barotrauma. Values shown are the mean effects (difference between means) and 95% confidence intervals. The average effect was calculated using a random-effects model.

### Oxygenation

Ten studies^(13,14,16,17,19,20–22,24,27)^ analyzed the impact of RM on oxygenation. A random-effects model was used (I²=99%, df=9, p<0.00001) in which there was a statistically significant difference between the RM and the Control Groups (difference between means=45.05; 95%CI=31.37-58.74) (Figure 2B).

### Barotrauma

Twelve studies^(13,14,16–21,23,24,26,27)^ analyzed the impact of RMs on the risk of barotrauma. A random-effects model was used (I²=51%, df=9, p=0.03), in which there was a statistically significant difference between the RM and the Control Groups (relative risk=0. 93; 95%CI=0.56-1.54) (Figure 2C).

## DISCUSSION

Analysis of the studies revealed that the RM had no significant effects on the mortality rate or barotrauma, even favoring the Control Group in some studies. The maneuver yielded positive results during oxygenation. None of the studies evaluated or mentioned whether alveolar recruitability was verified.

Cavalcanti et al.^(14)^ showed that pulmonary recruitment with PEEP titration over a 28-day period increased the mortality of patients with ARDS when compared to low levels of PEEP. Notably, the ART study^(14)^ was the only study with a high risk of bias. The study did not blind the professionals, even though the procedure was not simple. Huh et al.^(19)^ reported higher mortality rates because once the alveoli were recruited, an investigation was needed to obtain the correct levels of PEEP.

With regard to in-hospital mortality and barotrauma rates, Hodgson et al. reported that the use of recruitment with PEEP titration compared to the conventional mode was not relevant, although there was a PaO_2_/FiO_2_ ratio improvement in the first 24 h.^(18)^ Kung et al. reported that the barotrauma rates and mortality within 28 days were not significant but oxygenation improved on days 1 and 3.^(21)^

Maede et al. described RM with high PEEP levels due to the high risk of lung damage; however, it was observed that mortality rates from hypoxemia were reduced as the open lung strategy improved oxygenation.^(24)^ Lam et al.^(22)^ described a faster increase in the PaO_2_/FiO_2_ ratio from the 1^st^ to the 5^th^ of recruitment due to an improvement in lung compliance. A higher 28-day mortality rates for all causes were observed.

Amato et al. showed that the mortality rate was lower in the Intervention Group, which can be explained by the higher weaning rate and lower tidal volume used compared to the Control Group.^(13)^ Barotrauma rate was lower, and oxygenation was higher in the Intervention Group.^(13)^ Hodgson et al. did not find a statistically significant difference in the mortality rate, and there were no barotrauma events in either group, although oxygenation was higher in the Intervention Group.^(17)^

Constantim et al.^(16)^ observed that patients with ARDS do not have the same phenotypes; therefore, ventilatory parameters and the use of RM must be linked according to the individual characteristics of the patients. Improvements in oxygenation were also observed; however, due to incorrect classification, the mortality rates were worse compared to standard strategies.^(16)^ Chung et al.^(15)^ reported an improvement in oxygenation but no significant change in the mortality.

Kacmarek et al.^(20)^ found no significant differences in mortality and no barotrauma events in either group. Oxygenation levels were higher in the intervention group than in the Control Group.^(20)^ In the study by Xi et al.^(26)^ no significant difference was found in in-hospital mortality, only in intra-ICU mortality, which was lower in the intervention group, and oxygenation showed a significant increase in both groups, with no significant differences. Yu et al. also found no significant differences in mortality and barotrauma rates in both groups; however, there was a significant improvement in oxygenation in the Intervention Group.^(27)^

This study has some limitations, including small sample size, presence of heterogeneity in some studies, and limitations in the information provided, making it difficult to assess the methodological quality. More robust studies are required to evaluate other strategies for applying RMs.

## CONCLUSION

Recruitment maneuver is not a strategy to be used routinely in the care of patients with acute respiratory distress syndrome but serves as a good rescue strategy when other methods fail to improve oxygenation.

## SUPPLEMENTARY MATERIAL Recruitment maneuvers in patients with acute respiratory distress syndrome: a systematic review and metanalysis

Alice Cardoso de Jesus, Arthur Marchesini de Figueiredo, André Luiz Lisboa Cordeiro

**DOI: 10.31744/einstein_journal/2024RW0372**

### PUBMED STRATEGY

(("respiratory distress syndrome"[MeSH Terms] OR ("respiratory"[All Fields] AND "distress"[All Fields] AND "syndrome"[All Fields]) OR "respiratory distress syndrome"[All Fields] OR (("adult"[MeSH Terms] OR "adult"[All Fields] OR "adults"[All Fields] OR "adult s"[All Fields]) AND ("respiratory distress syndrome"[MeSH Terms] OR ("respiratory"[All Fields] AND "distress"[All Fields] AND "syndrome"[All Fields]) OR "respiratory distress syndrome"[All Fields] OR ("acute"[All Fields] AND "respiratory"[All Fields] AND "distress"[All Fields] AND "syndrome"[All Fields]) OR "acute respiratory distress syndrome"[All Fields])) OR (("human s"[All Fields] OR "humans"[MeSH Terms] OR "humans"[All Fields] OR "human"[All Fields]) AND ("respiratory distress syndrome"[MeSH Terms] OR ("respiratory"[All Fields] AND "distress"[All Fields] AND "syndrome"[All Fields]) OR "respiratory distress syndrome"[All Fields])) OR ("acute chest syndrome"[MeSH Terms] OR ("acute"[All Fields] AND "chest"[All Fields] AND "syndrome"[All Fields]) OR "acute chest syndrome"[All Fields]) OR ("respiratory distress syndrome"[MeSH Terms] OR ("respiratory"[All Fields] AND "distress"[All Fields] AND "syndrome"[All Fields]) OR "respiratory distress syndrome"[All Fields]) OR ("respiratory distress syndrome"[MeSH Terms] OR ("respiratory"[All Fields] AND "distress"[All Fields] AND "syndrome"[All Fields]) OR "respiratory distress syndrome"[All Fields] OR ("adult"[All Fields] AND "respiratory"[All Fields] AND "distress"[All Fields] AND "syndrome"[All Fields]) OR "adult respiratory distress syndrome"[All Fields]) OR ("respiratory distress syndrome"[MeSH Terms] OR ("respiratory"[All Fields] AND "distress"[All Fields] AND "syndrome"[All Fields]) OR "respiratory distress syndrome"[All Fields] OR ("shock"[All Fields] AND "lung"[All Fields]) OR "shock lung"[All Fields]) OR ("respiratory distress syndrome"[MeSH Terms] OR ("respiratory"[All Fields] AND "distress"[All Fields] AND "syndrome"[All Fields]) OR "respiratory distress syndrome"[All Fields] OR "ards"[All Fields]) OR (("adult"[MeSH Terms] OR "adult"[All Fields] OR "adults"[All Fields] OR "adult s"[All Fields]) AND ("respiratory distress syndrome"[MeSH Terms] OR ("respiratory"[All Fields] AND "distress"[All Fields] AND "syndrome"[All Fields]) OR "respiratory distress syndrome"[All Fields] OR "ards"[All Fields])) OR ("respiratory distress syndrome"[MeSH Terms] OR ("respiratory"[All Fields] AND "distress"[All Fields] AND "syndrome"[All Fields]) OR "respiratory distress syndrome"[All Fields] OR ("human"[All Fields] AND "ards"[All Fields]) OR "human ards"[All Fields])) AND "clinical trial"[Publication Type] AND (((("recruit"[All Fields] OR "recruited"[All Fields] OR "recruiter"[All Fields] OR "recruiters"[All Fields] OR "recruiting"[All Fields] OR "recruitment"[All Fields] OR "recruitments"[All Fields] OR "recruits"[All Fields]) AND ("maneuver"[All Fields] OR "maneuvered"[All Fields] OR "maneuvering"[All Fields] OR "maneuverings"[All Fields] OR "maneuvers"[All Fields] OR "manoeuvrability"[All Fields] OR "manoeuvrable"[All Fields] OR "manoeuvre"[All Fields] OR "manoeuvred"[All Fields] OR "manoeuvres"[All Fields] OR "manoeuvring"[All Fields])) OR (("alveolar"[All Fields] OR "alveolarization"[All Fields] OR "alveolars"[All Fields]) AND ("recruit"[All Fields] OR "recruited"[All Fields] OR "recruiter"[All Fields] OR "recruiters"[All Fields] OR "recruiting"[All Fields] OR "recruitment"[All Fields] OR "recruitments"[All Fields] OR "recruits"[All Fields]) AND ("maneuver"[All Fields] OR "maneuvered"[All Fields] OR "maneuvering"[All Fields] OR "maneuverings"[All Fields] OR "maneuvers"[All Fields] OR "manoeuvrability"[All Fields] OR "manoeuvrable"[All Fields] OR "manoeuvre"[All Fields] OR "manoeuvred"[All Fields] OR "manoeuvres"[All Fields] OR "manoeuvring"[All Fields])) OR (("alveolar"[All Fields] OR "alveolarization"[All Fields] OR "alveolars"[All Fields]) AND ("recruit"[All Fields] OR "recruited"[All Fields] OR "recruiter"[All Fields] OR "recruiters"[All Fields] OR "recruiting"[All Fields] OR "recruitment"[All Fields] OR "recruitments"[All Fields] OR "recruits"[All Fields])) OR (("lung"[MeSH Terms] OR "lung"[All Fields] OR "pulmonary"[All Fields]) AND ("recruit"[All Fields] OR "recruited"[All Fields] OR "recruiter"[All Fields] OR "recruiters"[All Fields] OR "recruiting"[All Fields] OR "recruitment"[All Fields] OR "recruitments"[All Fields] OR "recruits"[All Fields]) AND ("maneuver"[All Fields] OR "maneuvered"[All Fields] OR "maneuvering"[All Fields] OR "maneuverings"[All Fields] OR "maneuvers"[All Fields] OR "manoeuvrability"[All Fields] OR "manoeuvrable"[All Fields] OR "manoeuvre"[All Fields] OR "manoeuvred"[All Fields] OR "manoeuvres"[All Fields] OR "manoeuvring"[All Fields])) OR (("lung"[MeSH Terms] OR "lung"[All Fields] OR "pulmonary"[All Fields]) AND ("volum"[All Fields] OR "volume"[All Fields] OR "volumes"[All Fields] OR "voluming"[All Fields]) AND ("recruit"[All Fields] OR "recruited"[All Fields] OR "recruiter"[All Fields] OR "recruiters"[All Fields] OR "recruiting"[All Fields] OR "recruitment"[All Fields] OR "recruitments"[All Fields] OR "recruits"[All Fields])) OR (("lung"[MeSH Terms] OR "lung"[All Fields] OR "pulmonary"[All Fields]) AND ("recruit"[All Fields] OR "recruited"[All Fields] OR "recruiter"[All Fields] OR "recruiters"[All Fields] OR "recruiting"[All Fields] OR "recruitment"[All Fields] OR "recruitments"[All Fields] OR "recruits"[All Fields]))) AND "clinical trial"[Publication Type]) AND (("mortality"[MeSH Terms] OR "mortality"[All Fields] OR "mortalities"[All Fields] OR "mortality"[MeSH Subheading] OR ("hospital mortality"[MeSH Terms] OR ("hospital"[All Fields] AND "mortality"[All Fields]) OR "hospital mortality"[All Fields] OR "in hospital mortality"[All Fields]) OR (("intensive care units"[MeSH Terms] OR ("intensive"[All Fields] AND "care"[All Fields] AND "units"[All Fields]) OR "intensive care units"[All Fields] OR "icu"[All Fields]) AND ("mortality"[MeSH Terms] OR "mortality"[All Fields] OR "mortalities"[All Fields] OR "mortality"[MeSH Subheading])) OR ("barotrauma"[MeSH Terms] OR "barotrauma"[All Fields] OR "barotraumas"[All Fields]) OR ("cell respiration"[MeSH Terms] OR ("cell"[All Fields] AND "respiration"[All Fields]) OR "cell respiration"[All Fields] OR "oxygenation"[All Fields] OR "oxygen"[MeSH Terms] OR "oxygen"[All Fields] OR "oxygen s"[All Fields] OR "oxygenate"[All Fields] OR "oxygenated"[All Fields] OR "oxygenates"[All Fields] OR "oxygenating"[All Fields] OR "oxygenations"[All Fields] OR "oxygenative"[All Fields] OR "oxygenator s"[All Fields] OR "oxygenators"[MeSH Terms] OR "oxygenators"[All Fields] OR "oxygenator"[All Fields] OR "oxygene"[All Fields] OR "oxygenic"[All Fields] OR "oxygenous"[All Fields] OR "oxygens"[All Fields])) AND "clinical trial"[Publication Type])) AND (clinicaltrial[Filter])

### CENTRAL STRATEGY

Respiratory distress syndrome OR Adult acute respiratory distress syndrome OR Human respiratory distress syndrome OR Acute chest syndrome OR Respiratory distress syndrome OR Adult respiratory distress syndrome OR Shock lung OR ARDS OR Adult ARDS OR Human ARDS in Title Abstract Keyword AND Recruitment maneuvers OR Alveolar recruitment maneuver OR Alveolar recruitment OR Pulmonary recruitment maneuver OR Pulmonary volume recruitment OR Pulmonary recruitment in Title Abstract Keyord AND Mortality OR In-hospital mortality OR ICU mortality OR Barotrauma OR Oxygenation in Title Abstract Keyword - (Word variations have been searched)
